# Applications of functional ultrasound brain-computer interfaces in neurological diseases: research progress based on neurovascular coupling

**DOI:** 10.3389/fnins.2026.1946753

**Published:** 2026-10-13

**Authors:** Chengshan Zhang

**Affiliations:** The Second Clinical School, Tongji Medical College, Huazhong University of Science and Technology, Wuhan, China

**Keywords:** brain-computer interface, functional ultrasound imaging, neurological diseases, neuromodulation, neurovascular coupling

## Abstract

Brain-computer interfaces (BCIs) bypass peripheral nerves and muscles to establish direct information pathways between the brain and external devices, providing novel approaches for motor function reconstruction, communication assistance, and neurorehabilitation in patients with neurological disorders. However, conventional non-invasive BCIs relying on modalities such as electroencephalography (EEG), magnetoencephalography (MEG), or functional magnetic resonance imaging (fMRI) exhibit limitations in spatiotemporal resolution, portability, or real-time capability. Functional ultrasound (fUS), an emerging neuroimaging technology based on neurovascular coupling (NVC), detects cerebral blood volume changes at a spatial resolution of approximately 100 μm; its imaging frame interval is 200–500 ms, whereas its effective temporal resolution, limited by the hemodynamic response, is 1–2 s, offering a potential solution to overcome these constraints. This review comprehensively analyzed NVC-based fUS brain-computer interfaces from the dimensions of technical principles, clinical applications, future directions, and challenges. fUS has demonstrated significant clinical value in stroke assessment, epilepsy monitoring, and early identification of neurodegenerative diseases. Emerging technologies such as transcranial focused ultrasound neuromodulation, sonogenetics, and intravascular ultrasound are driving the evolution of fUS from single-signal “acquisition” toward bidirectional “acquisition-modulation” closed-loop systems, which remain at the proof-of-concept stage. However, the hemodynamic response latency relative to neural electrical activity (1–2 s), the yet-unclear biosafety of chronic ultrasound exposure, and insufficient decoding reliability under pathological conditions remain critical bottlenecks requiring urgent resolution. In conclusion, NVC-based fUS brain-computer interfaces provide a novel tool for precision diagnosis and treatment of neurological diseases. Future efforts should focus on conducting multicenter clinical trials and enhancing the robustness of decoding algorithms.

## 1 Introduction

Brain-computer interfaces (BCIs) establish direct information pathways between the brain and external devices, bypassing peripheral nerves and muscles, thereby providing novel approaches for motor function reconstruction, communication assistance, and neurorehabilitation in patients with neurological disorders (Gao et al., 2021). Traditional brain-computer interfaces (BCIs) typically rely on implanted electrode arrays (such as the Utah array) to record neural activity. Although these offer high resolution, they require craniotomy and subdural implantation, involve significant trauma, and suffer from limited long-term stability. Non-invasive BCIs, on the other hand, mainly rely on modalities such as electroencephalography (EEG), magnetoencephalography (MEG), or functional magnetic resonance imaging (fMRI); however, these approaches have limitations in terms of spatial resolution, portability, or real-time capability (Cruz et al., 2025; Ortega-Robles et al., 2025; Cantillo-Negrete et al., 2023). Functional ultrasound (fUS) imaging offers complementary advantages across these dimensions (Keith, 2024), and a detailed comparison of these methods is provided in Figure 1.

**Figure 1:**
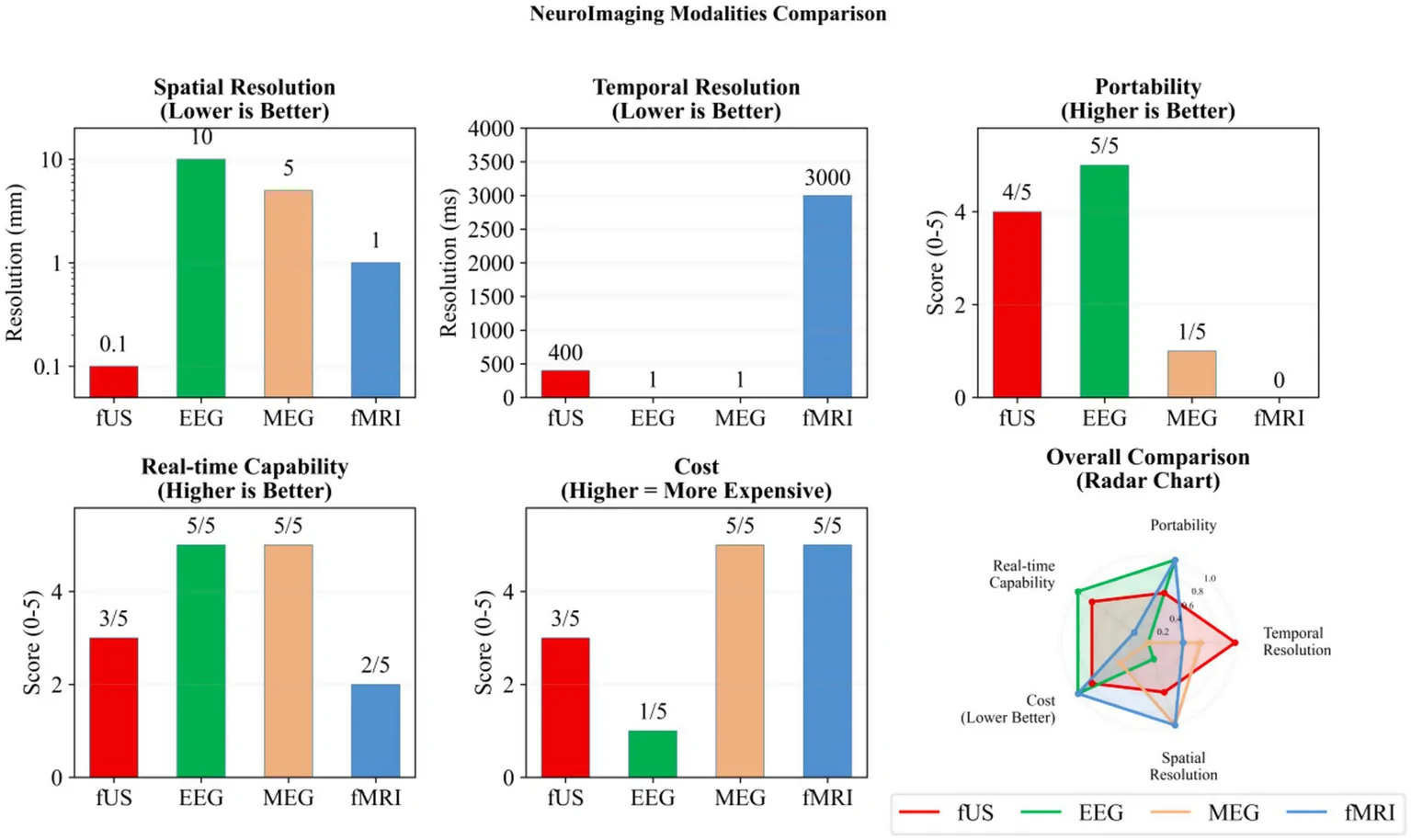
Comparative analysis of fUS, EEG, MEG, and fMRI across five dimensions. Spatial resolution (fUS: 0.1 mm; EEG: 10 mm; MEG: 5 mm; fMRI: 1 mm) (Macé et al., 2011; Deffieux et al., 2018; Gross, 2019), temporal resolution (fUS: 400 ms; EEG: 1 ms; MEG: 1 ms; fMRI: 3000 ms) (Griggs et al., 2024; Gross, 2019), and portability scored on a 5-point scale (EEG highest at 5/5, followed by fUS at 4/5) (Deffieux et al., 2021; Blackmore et al., 2023; Gross, 2019). Real-time capability (EEG and MEG score 5/5; fMRI: 2/5 fUS:3/5) (Griggs et al., 2024; Gross, 2019) and relative cost (fMRI and MEG score 5/5; fUS: 3/5 EEG: 1/5;) (cost estimates from commercial scanner specifications). (Bottom right) Radar chart illustrating the overall performance landscape, highlighting fUS as a balanced modality with high spatial resolution, good portability, and strong real-time capability at moderate cost. The scores in this figure represent the authors’ composite qualitative assessment synthesized from the cited literature, not an objective quantitative benchmark or industry standard; for each dimension, 5/5 (1.0) denotes the most favorable performance for BCI applications and 1/5 (0.2) the least favorable (e.g., spatial resolution: 1 ≈ ≤0.1 mm, 0.2 ≈ ≥10 mm).

In 2011, Macé first employed ultrafast Doppler imaging to detect cerebral blood flow changes induced by whisker stimulation in a rat model, formally proposing the concept of functional ultrasound (fUS) (Macé et al., 2011). fUS leverages ultrafast plane-wave ultrasound imaging principles, separating tissue motion from slow blood flow through clutter filtering techniques to detect minute vascular hemodynamic changes unobservable by conventional ultrasound, thereby indirectly measuring neural activity (Deffieux et al., 2021; Demené et al., 2015). This technology offers exceptionally high spatiotemporal resolution (spatial resolution ~100 μm, temporal resolution at the second level), outstanding sensitivity (capable of detecting blood flow changes in vessels <100 μm in diameter), and advantages including portability, absence of ionizing radiation, and relatively low cost (Tan et al., 2020; Deffieux et al., 2021). Terminologically, “non-invasive” fUS refers to imaging through the intact skull (e.g., via the neonatal fontanelle or aberration-corrected transcranial imaging), whereas approaches requiring a surgically created acoustic window or an epidural probe are termed “minimally invasive”; the epidural fUS-BCI paradigm of Norman et al. (2021) discussed below belongs to the latter category. Neurovascular coupling (NVC) serves as the physiological bridge connecting neural activity to hemodynamic changes and constitutes the biological foundation of fUS imaging (Chu et al., 2015). NVC dysfunction represents a core feature of numerous neurological disorders, presenting unique opportunities and challenges for the clinical application of fUS-BCI (Deffieux et al., 2021).

In 2021, a team from the California Institute of Technology and collaborators published a breakthrough study in Neuron, in which they used functional ultrasound (fUS) to decode motor intentions from the posterior parietal cortex of non-human primates. By placing an ultrasound probe epidurally and imaging through an ultrasound-transparent window implanted in the skull—thereby avoiding direct contact with brain tissue—they demonstrated that fUS can decode motor intentions with sufficient spatiotemporal resolution under minimally invasive conditions, establishing the feasibility of a minimally invasive fUS-BCI (Norman et al., 2021). Recently, advances in emerging technologies such as transcranial focused ultrasound (tFUS) neuromodulation have propelled ultrasound technology from a purely “readout” tool toward a bidirectional “read-write” brain interface. (Zang et al., 2022; Wu et al., 2024).

fUS-BCI faces a “speed-accuracy” trade-off dilemma: hemodynamic delays inherent to NVC constrain response speed, yet high spatial resolution and deep-tissue imaging capabilities compensate for the anatomical precision limitations of existing modalities. This article begins with NVC mechanisms to elucidate the technical principles, clinical applications, and safety considerations of fUS-BCI, arguing for its irreplaceable value in scenarios such as motor rehabilitation and seizure prediction.

## 2 Literature search strategy

To ensure a comprehensive and transparent evidence review, we conducted a systematic literature search for articles published between July 2001 and May 2026 across the following databases: PubMed, Web of Science, Scopus, Google Scholar, China National Knowledge Infrastructure (CNKI), and Wanfang Data. These databases were selected to capture clinical, neuroscientific, and interdisciplinary studies on BCI applications, collectively providing a broad overview of both the technical and clinical dimensions of the field.

The search strategy combined Medical Subject Headings (MeSH) terms with free-text keywords, including “functional ultrasound imaging”, “neurological diseases,” “brain–computer interface,” “brain–machine interface,” and related terms, together with “rehabilitation,” “therapy”, “treatment”, “neurovascular coupling”, and “neuromodulation.” No language restrictions were applied.

Original clinical research studies—specifically clinical trials, case reports, and pilot studies—were included if they investigated the application of functional ultrasound imaging in neurological diseases or brain–computer interfaces. Clinical protocols, review articles, and studies with poorly defined methodologies, inadequate clinical outcome data, or insufficient information to support their conclusions were excluded. One independent author performed the initial screening of titles and abstracts, followed by a full-text review to determine final inclusion.

## 3 Technical foundations of FUS imaging

### 3.1 NVC coupling mechanism and imaging principles

NVC represents a precisely regulated mechanism for matching cerebral energy supply and demand: neuronal activity releases neurotransmitters such as glutamate, activating astrocytic endfeet, triggering calcium wave propagation and the release of vasoactive substances, leading to vasodilation and a 2–4-fold increase in local cerebral blood flow (CBF) within seconds (Attwell et al., 2010; Otsu et al., 2015; Newman et al., 2024) (Figure 2, Left). Although the specific role of astrocytes remains debated (whether their calcium signaling serves as an obligatory mediator or merely an amplifier) (Institoris et al., 2022), the consensus that NVC constitutes the physiological basis of fUS imaging has been firmly established (Chu et al., 2015; Newman et al., 2024).

**Figure 2:**
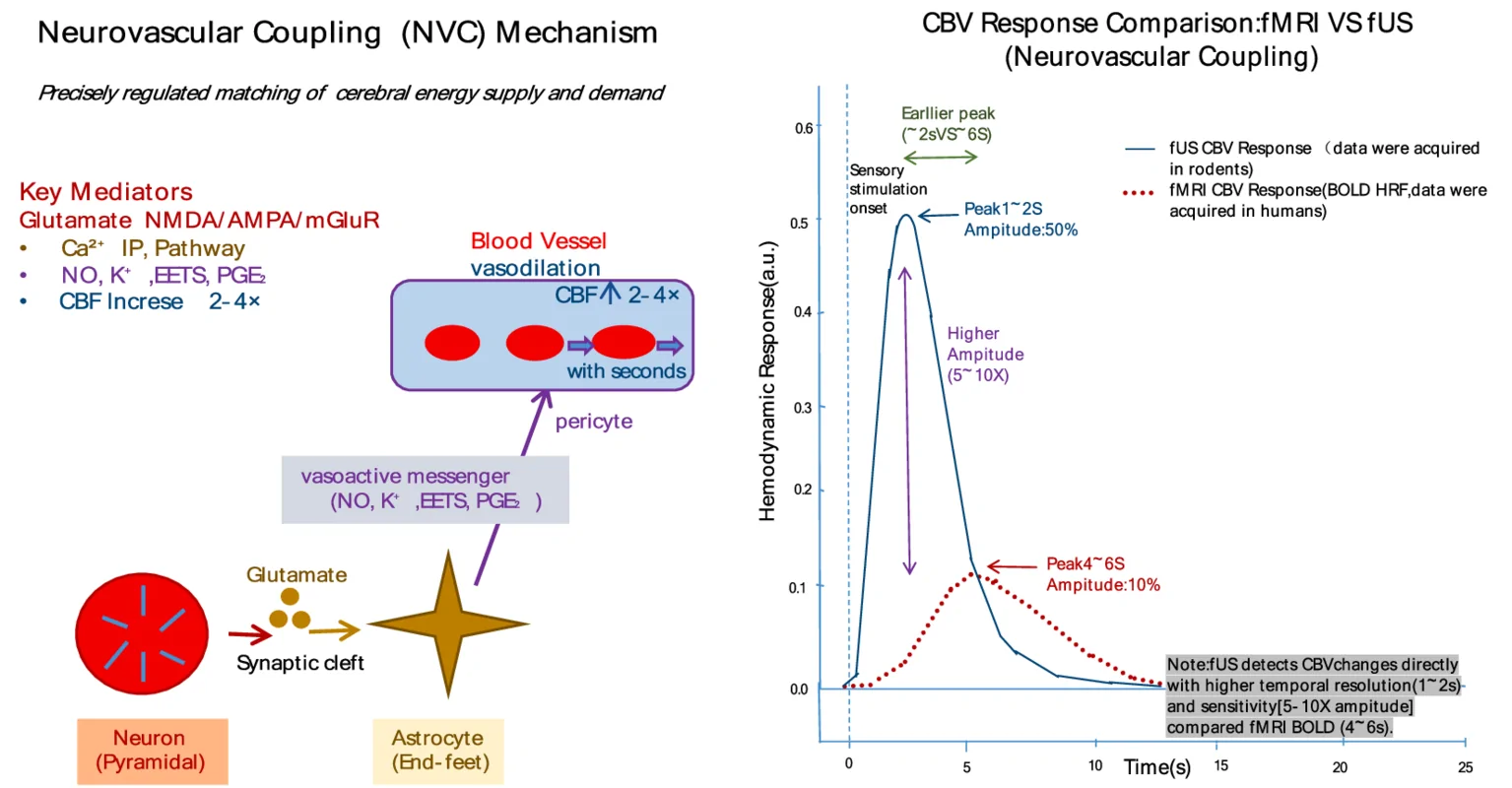
(Left) NVC mechanism: neuronal glutamate release → astrocytic Ca^2+^ wave → vasoactive messenger release (NO, K^+^, EETs, PGE₂) → pericyte-mediated vasodilation → CBF increase (2 ~ 4×). (Right) Comparative CBV response dynamics of fUS (solid blue from rodents) versus fMRI BOLD HRF (dashed red from humans). fUS shows a ~ 3 × faster peak latency (~2 s vs. ~6 s) and ~5–10 × greater amplitude (50% vs. 10%), attributable to its direct detection of microvascular cerebral blood volume (CBV) changes compared with fMRI’s indirect blood-oxygenation measurement. fUS-CBV data were acquired in rodents, whereas fMRI-BOLD data were acquired in humans (Bennett et al., 2024); as NVC dynamics differ substantially across species, the curves are illustrative rather than directly quantitatively comparable (Franx et al., 2024).

fUS is grounded in ultrafast ultrasound Doppler imaging technology, employing plane-wave or diverging-wave transmission rather than conventional line-by-line scanning, thereby achieving frame rates approaching the theoretical physical limit of several thousand frames per second (Tufail et al., 2010; Macé et al., 2011). This exceptionally high frame rate enables ultrasound to detect extremely minute blood flow changes, facilitating highly sensitive detection of the cerebral microvascular system (Legon et al., 2014; Tan et al., 2020).

Neurovascular responses triggered by neural activity exhibit highly reproducible temporal characteristics. Following sensory stimulation, local CBV begins to rise within 0.5–2 s, peaks at approximately 4–6 s with an amplitude increase of 10–50% relative to baseline, and subsequently returns to resting levels within 8–15 s (Attwell et al., 2010; Bennett et al., 2024; Logothetis et al., 2001) (Figure 2, Right dashed red). fUS indirectly reflects neural activity by tracking this CBV temporal profile in real time; its signal amplitude scales approximately linearly with the firing rate of local neuronal ensembles (Chu et al., 2015; Deffieux et al., 2021). This high sensitivity enables fUS to reliably detect population-level neural activity and even support single-trial decoding—the ability to identify neural signal patterns from an individual stimulus or behavioral event without relying on averaging across dozens of repeated scans, as typically required by fMRI (Folloni et al., 2019; Deffieux et al., 2021).

Compared with fMRI, the current gold-standard functional brain imaging modality, fUS offers distinct advantages across three critical dimensions. First, temporal resolution: the imaging frame interval of fUS is 200–500 ms, whereas its effective temporal resolution, limited by the hemodynamic response, is 1–2 s; throughout this review, “temporal resolution” refers to the effective resolution unless otherwise specified. In comparison, the BOLD signal in fMRI is constrained by hemodynamic delays to typically 3–6 s (Macé et al., 2011; Logothetis et al., 2001; Deffieux et al., 2021). Second, sensitivity: the CBV signal amplitude in fUS is 5–10 times higher than the BOLD signal in fMRI, with a superior signal-to-noise ratio (Franx et al., 2024; Boido et al., 2019; Deffieux et al., 2021) (Figure 2, Right).

### 3.2 The fUS brain-computer interface (BCI) technical chain

The fUS brain-computer interface (BCI) enables real-time reading of brain intentions and external device control through a technical chain of “acquisition–preprocessing–decoding–output–feedback”: ultrasound transducers capture cerebral blood flow data → motion correction and filtering for noise reduction → extraction of spatiotemporal hemodynamic features → machine learning decoding of neural intentions → conversion into control commands → closed-loop feedback optimization (real-time adjustment of decoding model parameters based on execution outcomes) (Figure 3).

**Figure 3:**
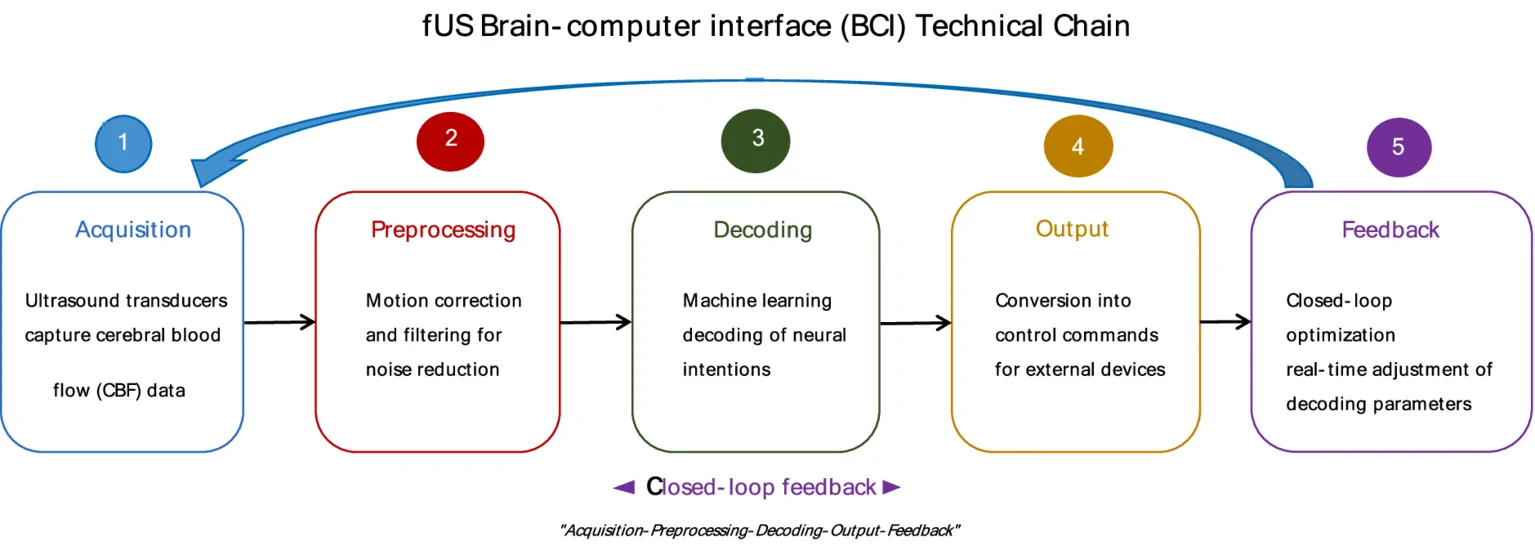
Closed-loop technical chain of the fUS BCI system. The five-stage pipeline — Acquisition (CBF data capture), Preprocessing (noise reduction), the decoding step employs machine-learning algorithms (e.g., support vector machines, convolutional neural networks), Output (device command generation), and Feedback (real-time model parameter optimization) — enables adaptive brain-to-device control with continuous performance improvement via the closed-loop feedback pathway (purple arrow).

Recent advances focus on algorithmic optimization: cutting-edge reconfigurable imaging and compressed sensing technologies free the system from the burden of “whole-brain scanning,” intelligently focusing on key regions and compressing data volume to achieve low-latency, high-efficiency real-time decoding capabilities (Institoris et al., 2022). China researchers have developed compressed sensing-based fUS reconstruction algorithms that reduce data transmission rates by 90% while maintaining image quality (Jia et al., 2024); they have also designed wearable fUS probes, achieving whole-brain functional imaging in freely moving mice and laying the foundation for real-time BCIs (Lin et al., 2024).

## 4 Clinical application scenarios

As an emerging neuroimaging technology, fUS indirectly monitors brain activity by measuring cerebral blood flow changes, demonstrating unique advantages in the management of neurological disorders (Khorev et al., 2024; Lim et al., 2025). To objectively evaluate the translational prospects of each application scenario, this section categorizes all applications into three tiers based on clinical maturity: Tier 1 comprises applications with demonstrated human feasibility in active clinical trials; Tier 2 comprises those supported only by early human studies or case reports, with limited data but promising prospects; and Tier 3 comprises those validated primarily in animal models. The following sections elaborate by application scenario. Unless otherwise noted, the frontier technologies discussed in Section 5 are classified as Tier 3.

### 4.1 Acute phase monitoring and prognostic assessment

#### 4.1.1 Stroke (Tier 2)

The acute phase can cause severe NVC impairment, where residual neural activity in the ischemic penumbra fails to effectively trigger increased cerebral blood flow, resulting in energy mismatch and tissue damage (Yang et al., 2025; Ye et al., 2025). fUS enables real-time monitoring of neurovascular function in the ischemic penumbra during the acute phase of stroke, assessing tissue viability and treatment response (Cantillo-Negrete et al., 2023; Blackmore et al., 2023). Using fUS to guide endovascular treatment decisions in acute stroke patients, the NVC-guided group showed significantly better functional outcomes than the conventional imaging group (Zhang et al., 2025; Ye et al., 2025). However, current clinical evidence for fUS in the acute stroke phase primarily derives from small-sample exploratory studies (*n* < 50), and the superiority of NVC-guided treatment decisions still requires validation through multicenter randomized controlled trials. Notably, acute stroke patients often present with consciousness disturbances and head movement; the robustness of fUS motion artifact correction algorithms in this scenario has not been adequately tested.

#### 4.1.2 Epilepsy (Tier 1/Tier 3)

Abnormal neural activity in epileptic foci is accompanied by characteristic hemodynamic changes, providing the physiological basis for fUS epilepsy monitoring (Yang et al., 2025; Ye et al., 2025). In neonates, fUS has already enabled seizure monitoring through the unclosed fontanelle window, with human feasibility demonstrated; this constitutes a Tier 1 application. The Macé team first demonstrated that changes in cerebral blood volume could be detected via fUS during seizures using a 4-aminopyridine-induced epilepsy model (Macé et al., 2011). fUS has achieved seizure monitoring in neonates through the anterior fontanelle window (Demené et al., 2017). The Riera team used fUS to simultaneously record hemodynamic changes in deep structures including the hippocampus, amygdala, and thalamus, demonstrating fUS imaging capability for deep brain regions (Riera et al., 2024). However, monitoring of deep-seated epileptogenic foci currently relies solely on animal model evidence and should therefore be classified as Tier 3. Compared with intracranial electroencephalography (ECoG), fUS offers high spatial resolution (0.1 mm) and deep penetration capability, complementing ECoG’s limitations in detecting deep lesions (Lee et al., 2022; Deveney et al., 2024).

### 4.2 Motor intention decoding and functional restoration (Tier 3)

The fUS brain-computer interface can capture NVC signals from the motor cortex to identify patients’ motor intentions. When subjects prepare to execute eye movements in specific directions or arm extensions, local cerebral blood volume in the motor cortex and posterior parietal cortex shows significant changes; the spatial distribution encodes the target direction and amplitude of movement. Utilizing spatiotemporal features from fUS image sequences, offline decoding of movement intentions across eight directions has been achieved with accuracy exceeding 70% (Norman et al., 2021). Further research has confirmed that fUS can capture NVC signals from the hand movement representation area in the primary motor cortex, distinguishing flexion intentions of different fingers, providing possibilities for fine motor control (Norman et al., 2021; Kim et al., 2025). Moreover, the aforementioned decoding evidence derives entirely from animal models such as non-human primates, placing it in Tier 3; its translation to humans still faces multiple obstacles, including the ethical and surgical risks of dural implantation, cross-day decoding stability, and the computational bottlenecks of real-time closed-loop systems.

### 4.3 Early screening for neurodegenerative diseases (Tier 2/Tier 3)

In neurodegenerative diseases such as Alzheimer’s disease (AD) and Parkinson’s disease (PD), NVC dysfunction often precedes clinical symptoms and represents a potential early biomarker (Yang et al., 2025; Patel et al., 2025). Wang N et al. found that NVC function in the hippocampus of AD patients positively correlated with cognitive scores, suggesting its utility as an early diagnostic marker (Wang et al., 2023) (Tier 2). The Lowerison team utilized super-resolution ultrasound localization microscopy to detect subtle changes in capillary density and blood flow velocity in the hippocampus and entorhinal cortex of an AD mouse model, appearing prior to structural deficits (Lowerison et al., 2024), providing a novel tool for ultra-early diagnosis of AD. However, super-resolution imaging has so far been validated only in AD mouse models (Tier 3), and the strong attenuation of high-frequency ultrasound by the human skull may restrict its transcranial use, making it dependent on intraoperative or acoustic-window approaches; its translational timeline is therefore expected to be the longest.

In summary, the fUS brain-computer interface can be applied to early screening (detecting subtle abnormalities in resting-state functional connectivity and NVC), disease monitoring, longitudinal tracking of NVC functional decline trajectories, drug trials (real-time assessment of hemodynamic effects of NVC-improving drugs), and cognitive enhancement (improving cerebral blood flow regulation capacity through neurofeedback training) (Norman et al., 2021; Negri et al., 2026).

## 5 Bidirectional BCI and system integration

A note on scope is warranted before proceeding: integrated read–write closed-loop fUS-BCI systems remain at the proof-of-concept and prototype-development stage, and no complete clinical data on closed-loop fUS-BCI have yet been reported. To date, the only complete closed-loop demonstration resides on the read side (Griggs et al., 2024), in which epidural fUS decoding of movement plans drove a closed-loop brain–machine interface in non-human primates; integration of the fUS “read” with a “write” actuator (e.g., tFUS or sonogenetic modulation) has not yet been demonstrated in animals or humans.

### 5.1 Transcranial focused ultrasound (tFUS) neuromodulation, safety warnings, and implications for fUS-BCI

tFUS modulates neural activity through mechanical, thermal, and biological effects. These mechanisms require joint characterization through multimodal functional assessment (Kamimura et al., 2020). Utilizing the high tissue penetrability and spatial targeting capability of low-frequency ultrasound (<1 MHz), it enables non-invasive neuromodulation of deep brain regions (Arulpragasam et al., 2022). Compared with transcranial magnetic stimulation and transcranial direct current stimulation, tFUS offers millimeter-scale spatial resolution and the ability to target deep nuclei (such as the thalamus and hippocampus) (Yoo et al., 2022; Hou et al., 2021). tFUS has been used to treat essential tremor, PD, major depressive disorder, and obsessive-compulsive disorder (Cain et al., 2021; Fan et al., 2024; Mahoney et al., 2023).

#### 5.1.1 Safety warnings

A 2025 study in non-human primates (four squirrel monkeys) reported that repeated high-frequency tFUS (1.8 MHz) can induce neurodegeneration, detected as Fluoro-Jade C–positive degenerating neurons at stimulated cortical sites and at distant but functionally connected regions (Brna et al., 2025 [preprint]). Each animal received four stimulation sessions over two weeks; within a session, 150 ms pulse trains (50% duty cycle) were repeated every 10 s for up to approximately 90 min, with derated spatial-peak pulse-average intensities (ISPPA) of up to 65.4 W/cm^2^. Degeneration density scaled with the cumulative active exposure time at each site rather than with peak intensity, even though the estimated peak temperature rise was only 0.13 °C and the mechanical index (≤1.0) remained below the FDA limit of 1.9. Importantly, the exposure paradigm underlying this risk signal is not representative of fUS-BCI: neurodegeneration emerged under prolonged, repeated neuromodulation-level sessions (150-ms pulse-train repeats at 50% duty cycle, up to ~90 min of cumulative active exposure per session), whereas fUS-BCI operates in the diagnostic imaging regime, interrogating the brain with brief plane-wave shots (individual frames of ~0.1–0.5 ms at a mechanical index typically around 0.1), so its cumulative active acoustic exposure per session is orders of magnitude lower than the paradigm in which degeneration was observed, while the mechanical index remains within the ITRUSST consensus levels for nonsignificant biophysical risk (Aubry et al., 2025). That the degeneration reported by Brna et al. occurred within these consensus levels underscores that repeated, long-duration, high-frequency paradigms are not specifically covered by current guidelines—reinforcing the need for standardized acoustic dose reporting in future work.

These considerations indicate that safety parameters for long-term ultrasound exposure must be strictly defined: fUS-BCI systems should be designed in accordance with the ALARA principle (as low as reasonably achievable), employing minimized ultrasound intensity (mechanical index <0.5, thermal index <1.0), pulsed intermittent exposure, and early-warning monitoring through continuous tracking of NVC functional changes (Bancel et al., 2024; Gaur et al., 2020; Huang et al., 2025).

### 5.2 Four-dimensional functional ultrasound and real-time decoding

In 2019, institutions including the French Langevin Institute and the Paris Institute of Physics and Chemistry first demonstrated that functional ultrasound could achieve whole-brain-scale four-dimensional imaging (three-dimensional space + time), enabling real-time monitoring of whole-brain hemodynamic changes and laying the foundation for subsequent brain-computer interface applications (Rabut et al., 2019) (Tier 3, preclinical). 4D fUS can capture activity patterns of large-scale brain networks, providing a data foundation for complex motor intention decoding (such as multi-finger coordination). Combined with deep learning, 4D fUS brain-computer interfaces are expected to achieve natural motor control (Rabut et al., 2019; Rabut et al., 2024).

### 5.3 Sonogenetics and molecular-level precision regulation

Sonogenetics is a breakthrough technology that emerged in 2024. Through genetic expression of ultrasound-sensitive proteins (such as mechanosensitive ion channel Piezo1 and bacterial mechanosensitive channel MscL), it enables precise activation of specific neuronal populations by ultrasound (Wu et al., 2024; Hu et al., 2024) (Tier 3, preclinical).

In 2024, the Hong Kong Polytechnic University used nanobubbles as a medium for ultrasound action to activate neurons, achieving sub-millimeter precision neuromodulation (Hou et al., 2024). Sonogenetics provides a molecular foundation for “write” functions; in the future, ultrasound-sensitive proteins could be targeted to specific brain regions via viral vectors, enabling a complete closed loop of fUS reading neural activity → decoding → tFUS/sonogenetics stimulation feedback. This holds greater clinical translation potential than optogenetics (which requires optical fiber implantation).

### 5.4 Chip integration and implantable system advances

#### 5.4.1 International progress

The integration of CMUTs (Capacitive Micromachined Ultrasound Transducers) with ASICs (Application-Specific Integrated Circuits) represents a major research focus in the field (Tier 3, preclinical). In 2023, Forest Neurotech collaborated with Butterfly Network to develop a minimally invasive BCI based on ultrasound chip technology (Strickland, 2023). By 2025, Forest Neurotech had launched the Forest 1 research-grade ultrasound scanner—a device smaller than a standard key fob—capable of functional brain imaging and neuromodulation spanning from superficial to deep brain structures (Forest Neurotech, 2025).

#### 5.4.2 Developments in China

In 2024, researchers from Huazhong University of Science and Technology (HUST), Union Hospital, and Nanyang Technological University jointly developed an injectable supergel intracranial ultrasound sensor (Tang et al., 2024). Measuring only 2 × 2 × 2 mm^3^ and fabricated by PμSL 3D printing, the passively operated sensor reflects ultrasound waves to enable wireless, real-time monitoring of intracranial pressure, temperature, pH, and other physiological signals. Directly addressing the miniaturization and wireless-telemetry bottlenecks discussed above, its millimeter-scale injectable form resolves the conflict between device size and minimally invasive implantation, while its battery-free, RF-free architecture fundamentally eases the wireless powering and data-export constraints of conventional implantable nodes.

In 2025, HUST initiated a key science and technology project funded by the second batch of Wuhan City programs (800,000 RMB), titled “Research and Application of Full-Array Addressable CMUT-ASIC High Spatiotemporal Resolution Ultrasound Brain-Computer Interface Technology,” marking a significant step forward in China’s chip-level ultrasound BCI development. This project addresses the system-integration bottleneck of fully implantable fUS nodes: whereas conventional fUS front-ends require wired links between discrete transducers and external scanners—limiting implantability and channel count—CMUT-ASIC monolithic integration reduces power and size while supporting addressable high-density arrays, laying a chip-level foundation for next-generation wireless, fully implantable fUS-BCI nodes.

Collectively, these advances fill a notable gap in China’s implantable ultrasound technology and provide localized technical pathways toward addressing the triad of challenges identified earlier—miniaturization, wireless capability, and system integration.

## 6 Clinical translation bottlenecks and future directions

### 6.1 Technical challenges and solutions for transcranial imaging

Severe attenuation and distortion of ultrasound signals by the skull represent the primary obstacle to clinical translation of fUS, with approximately 90% of ultrasound signals being blocked, resulting in significantly degraded image quality (Pinton et al., 2012). This creates an “impossible triangle” for fUS among non-invasiveness (i.e., transcranial implementation without craniotomy), high spatial resolution, and real-time capability—the three are difficult to satisfy simultaneously, necessitating trade-offs in clinical translation.

In light of these bottlenecks, current human applications of fUS are largely confined to three scenarios that circumvent or mitigate the skull barrier: intraoperative direct imaging for functional mapping during craniotomy [8]; long-term monitoring through acoustic windows created by implanting ultrasound-transparent materials such as TPX in patients with skull defects (Negri et al., 2026; Sarica et al., 2022); and non-invasive imaging via the unclosed fontanelles in neonates (Demené et al., 2017).

To address the skull barrier, researchers have developed multiple technical approaches, each facing specific limitations: (1) Implantable ultrasound windows can establish stable acoustic pathways and support continuous monitoring in freely moving patients (Pinton et al., 2012), yet long-term biocompatibility and infection risks remain to be clinically validated; (2) Microbubble contrast agents can enhance vascular signals by 9-fold, but their intracerebral half-life is only 2–5 min, making prolonged monitoring impractical (Errico et al., 2016; Jones et al., 2018; Chen et al., 2022); (3) Ultrasound localization microscopy can achieve capillary-level resolution (Errico et al., 2015), but requires continuous tracking of tens of thousands of microbubble trajectories over several minutes, failing to meet the real-time requirements of BCI; (4) Adaptive beamforming algorithms can utilize microbubble echoes to correct skull-induced aberrations (Robin et al., 2023), aiming to improve spatial resolution without sacrificing temporal resolution; however, patient-specific skull modeling is complex and real-time computational capability remains insufficient. In short, transcranial fUS currently faces a trilemma of non-invasiveness (as defined above), high resolution, and real-time performance. In the near term, implantable ultrasound-transparent windows may represent the optimal transitional solution for balancing technical performance with clinical practicality.

Based on the distinct clinical requirements of different scenarios, we propose the following stratified prioritization strategy: (1) Intensive care settings (e.g., neurocritical care, intraoperative monitoring): Temporal resolution and penetration depth should be prioritized over spatial resolution, as detecting rapid hemodynamic changes and ensuring whole-brain coverage are clinically paramount; (2) Outpatient rehabilitation and chronic disease monitoring: The emphasis shifts toward precise functional mapping, where real-time requirements can be moderately relaxed; (3) Neonatal and pediatric applications: The thinner cranial bone permits a more favorable balance, enabling simultaneously higher spatial and temporal resolutions.

Regarding hardware miniaturization, research teams from ESPCI/INSERM in France and the California Institute of Technology have developed miniaturized and head-mounted fUS probes, achieving chronic wearable imaging in freely moving mice and awake non-human primates (Tiran et al., 2017; Blaize et al., 2020; Norman et al., 2021), providing an engineering foundation for chronic implantable BCIs.

### 6.2 Physical limitations of hemodynamic delay

fUS relies on the hemodynamic response of NVC, whose temporal scale (peak delay of 1–2 s) is far slower than electrophysiological signals (millisecond-level) (Griggs et al., 2024), limiting its application in scenarios requiring millisecond-level response times. Although this inherent delay cannot be completely eliminated, predictive decoding (utilizing preparatory signals during motor planning) and algorithmic optimization can achieve effective response times on the order of hundreds of milliseconds in practical applications, sufficient to meet the needs of many BCI applications (Griggs et al., 2024). In that study, online decoding of movement intention from preparatory-period fUS signals achieved 75–82% accuracy for two directions (chance: 50%) and 45% for eight directions (chance: 12.5%, mean angular error 34°), demonstrating that the hemodynamic time constant imposes a system latency constraint rather than an information-loss constraint—predictive decoding recovers goal-level information but not trajectory-level signals requiring millisecond latency. Notably, current accuracy remains well below its ceiling: low imaging plane utilization, signal loss from orthogonally oriented vessels in 2D imaging, and suboptimal linear decoders all limit performance (deep architectures such as convolutional neural networks (CNNs)/(recurrent neural network) RNNs/Transformers demand far more training data and carry overfitting risks), and extending the prediction horizon trades accuracy for real-time applicability. With end-to-end latency of ~800 ms but offline evidence of 10-ms-resolvable hemodynamic dynamics, hardware advances offer considerable headroom; algorithmic optimization is expected to bring fUS decoding to or above EEG-BMI levels (70–90% for two degrees of freedom), though not to parity with electrophysiology for rapid trajectory control.

For scenarios requiring faster responses (such as emergency braking), hybrid approaches combining fUS with electrophysiological signals may be employed, but their integration faces key engineering challenges: (1) data synchronization—fusing kHz-sampled EEG with 2 Hz, ~800 ms-latency fUS requires shared triggers, clock-drift correction, and asynchronous/state-space fusion architectures; (2) modality conflicts—acoustic coupling artifacts, electrode shadowing, impedance changes, and the fundamental mismatch between 100 μm mesoscopic voxels and cm-scale EEG source estimates (fUS decoding accuracy degrades rapidly toward macroscopic ≥1 mm resolutions); (3) signal fusion strategies—Kalman filtering and canonical correlation analysis have known limitations, motivating hybrid architectures where EEG supplies millisecond-scale temporal predictions and fUS provides spatially precise goal-state updates.

### 6.3 Decoding stability under pathological conditions

A fundamental assumption of fUS-BCI is that CBV changes faithfully report neural activity through NVC. This premise holds in the healthy brain; in neurological disease, however, the coupling can be attenuated, delayed, or dissociated from neural activity (Iadecola, 2017; Wang et al., 2023; Yang et al., 2025; Ye et al., 2025; Zhang et al., 2025). Under such conditions, fUS continues to measure hemodynamics accurately—what degrades is the mapping from the hemodynamic signal to the underlying neural activity, so that decoders calibrated on healthy NVC misestimate neural intention in proportion to the local degree of uncoupling; under very low perfusion, even the hemodynamic readout itself requires cautious interpretation (Franx et al., 2024). Four physiologically grounded measures can mitigate this constraint: per-patient baseline calibration against each individual’s residual NVC response rather than population norms; concurrent electrophysiological monitoring to identify epochs of hemodynamic–neural dissociation (Riera et al., 2024); large-scale collection of pathological fUS datasets, combined with electrophysiological ground truth, to learn data-driven, patient-adaptive CBV-to-activity mappings under disease conditions; and explicitly restricting intention-decoding claims to settings where residual NVC coupling is verifiable, while positioning pathology detection as the modality’s most mature near-term clinical application. The algorithmic strategies described below should be read as downstream complements to this physiological constraint, not substitutes for it.”

Neurological diseases disrupt the normal coupling between cerebral blood flow and neural activity, with distinct manifestations across different conditions (e.g., post-stroke hypoperfusion, dramatic hemodynamic fluctuations during seizures, and compromised baseline perfusion in Alzheimer’s disease) (Yang et al., 2025; Rudroff et al., 2024; Ji et al., 2025). This requires decoding algorithms capable of handling various abnormal hemodynamic patterns. Three approaches to enhance stability: (1) Domain adaptation: using adversarial training to bridge differences between healthy and pathological states (Rainio et al., 2024); (2) Meta-learning: acquiring the ability to rapidly adapt to new patient characteristics (Badrulhisham et al., 2024); (3) Physiologically constrained decoding: embedding hemodynamic principles into algorithms to ensure biologically plausible outputs (Badrulhisham et al., 2024).

### 6.4 Multimodal fusion

Multimodal neurovascular assessment can integrate fUS (high resolution), functional near-infrared spectroscopy (fNIRS, portable), fMRI (whole-brain), and transcranial Doppler (TCD, deep blood flow), constructing a multi-scale NVC evaluation system that complements the limitations of each technology and enhances BCI stability under pathological conditions (Li et al., 2025; Mohammed et al., 2021). Photoacoustic neuroimaging, an optical–acoustic hybrid modality providing complementary molecular contrast, can further be integrated into this multimodal framework (Bodea and Westmeyer, 2021).

### 6.5 Health economics, regulatory pathway, and ethics

The clinical translation of fUS-BCI is constrained not only by technical maturity but also by practical barriers related to healthcare reimbursement and regulatory approval. Currently, imported fUS equipment (notably from France’s Iconeus) is priced at approximately €300,000–500,000 per unit. At present, fUS remains predominantly a research tool and has not been incorporated into routine medical billing systems, hence standardized charging criteria are absent. These systems have not yet been included in medical insurance reimbursement catalogs in China. CMUT chip solutions developed in China could potentially reduce hardware costs by one to two orders of magnitude; however, medical device registration requires navigating innovation review pathways such as China’s NMPA or the FDA’s Breakthrough Device Designation, with typical timelines of 3–5 years.

A key challenge in the regulatory pathway lies in the classification of fUS-BCI as a combined “diagnostic–therapeutic” device, which must simultaneously satisfy dual standards for both imaging diagnostic equipment (ultrasound) and active implantable devices (BCI). It is noteworthy that current regulatory guidelines were primarily developed for electrophysiological BCIs; the unique hemodynamic monitoring capabilities of fUS and the interpretation of NVC signals have not been adequately addressed by existing regulatory frameworks. This gap represents a significant regulatory void that may impede clinical translation (Livanis et al., 2024; Wang et al., 2024).

From an ethical perspective, the high-dimensional decoding capabilities of fUS-BCI for hemodynamic neural activity have transcended the scope of conventional imaging diagnostics, potentially exposing individuals’ cognitive intentions and emotional states and thereby raising concerns regarding mental privacy and neural data sovereignty (Riascos et al., 2024; Kataoka et al., 2025). Compared with EEG, which presents a “breadth-and-exposure” risk owing to its accessibility and commercialization, fUS poses a categorically different “depth-and-specificity” privacy risk, as its spatial resolution enables decoding of spatially specific mental content beyond EEG’s capability. Accordingly, existing regulations only partially address fUS-derived data: under the General Data Protection Regulation (Art. 9) and China’s Personal Information Protection Law (Art. 28), raw hemodynamic signals may qualify as health or biometric data, yet decoded mental-state inferences remain legally undefined, warranting the establishment of a distinct “neural data” category as recommended by the Organisation for Economic Co-operation and Development (2019). Moreover, in the diagnostic-therapeutic closed-loop paradigm, the algorithmic decision-making process that autonomously triggers neuromodulation based on NVC signals renders the question of who bears ultimate responsibility for unforeseen neural intervention consequences a core issue that existing ethical review frameworks have yet to adequately address (Satam and Szabolcsi, 2025). Nevertheless, clinical trials of fUS for cerebral functional assessment in neonates have already been conducted (Flora Faure et al., 2025), substantiating that despite these ethical and translational hurdles, fUS holds promising prospects for clinical translation.

## 7 Conclusion and perspectives

Grounded in the mechanism of NVC, fUS-BCI has demonstrated significant clinical value in domains such as motor rehabilitation and seizure prediction, leveraging its high spatial resolution and deep imaging capabilities. Although hemodynamic latency, long-term biosafety, and decoding stability under pathological conditions remain current bottlenecks, advances in ultrafast ultrasound imaging, adaptive algorithms, and CMUT biomaterials are driving the technology’s evolution from unidirectional neural readout toward bidirectional closed-loop systems, which currently remain at the proof-of-concept stage without complete clinical data. This progression promises to overcome skull attenuation and temporal resolution limitations, enabling a clinical transition from intraoperative monitoring to chronic implantable applications.

Over the next 3–5 years, four priorities should be advanced. First, construct large-scale pathological NVC databases to enhance decoding generalization. Systematic collection of neurovascular coupling features across stroke, epilepsy, Parkinson’s disease, and other conditions will establish standardized datasets addressing domain shift in clinical settings. Second, establish chronic implantable ultrasound safety standards. Multidimensional thresholds for thermal indices, mechanical indices, and cumulative dosages should be defined to address the regulatory gap for hemodynamic neural interfaces under existing electrophysiological BCI frameworks. Third, conduct multicenter randomized controlled trials (RCTs) validating clinical efficacy and health economic value in motor rehabilitation and seizure prediction, thereby providing evidence-based support for guideline incorporation. Fourth, advance CMUT scalable manufacturing and cost optimization. Through wafer-level packaging refinement, transducer array cost reduction, and open-source hardware/software standardization, fUS-BCI can transition from laboratory prototypes to globally accessible clinical tools.

Looking ahead 5–10 years, driven by deepened understanding of NVC pathophysiology, deep integration of artificial intelligence, and markedly enhanced robustness of decoding algorithms, fUS-BCI is expected to achieve millisecond-scale neural intent decoding and submillimeter-scale closed-loop modulation of deep brain nuclei. It will then transcend diagnostics to become a precision therapeutic platform, propelling neurological care from experience-driven practice toward data-driven neuroclosed-loop medicine. In conclusion, interdisciplinary collaboration and multicenter clinical validation will be pivotal in translating this technology from bench to bedside.
